# Enhancing bioprocessing of red pigment from immobilized culture of gamma rays mutant of the endophytic fungus *Monascus ruber *SRZ112

**DOI:** 10.1186/s13036-024-00439-y

**Published:** 2024-08-15

**Authors:** El-Sayed R. El-Sayed, Shaimaa A. Mousa, Tomasz Strzała, Filip Boratyński

**Affiliations:** 1 Department of Food Chemistry and Biocatalysis, Wrocław University of Environmental and Life Sciences, Norwida 25, Wrocław, 50-375 Poland; 2 Plant Research Department, Nuclear Research Center, Egyptian Atomic Energy Authority, Cairo, Egypt; 3 Department of Genetics, Wrocław University of Environmental and Life Sciences, Ul. Kożuchowska 7, Wrołcaw, 51-631 Poland

**Keywords:** Red pigment, *Monascus ruber*, Endophytic fungi, Immobilization, Gamma Radiation Mutagenesis, Response Surface Methodology

## Abstract

Considerable attention has been paid to exploring the biotechnological applications of several *Monascus* sp. for pigment production. In this study, our focus is on enhancing the bioprocessing of red pigment (RP) derived from the endophytic fungus *Monascus ruber* SRZ112. To achieve this, we developed a stable mutant strain with improved productivity through gamma irradiation. This mutant was then employed in the immobilization technique using various entrapment carriers. Subsequently, we optimized the culture medium for maximal RP production using the Response Surface Methodology. Finally, these immobilized cultures were successfully utilized for RP production using a semi-continuous mode of fermentation. After eight cycles of fermentation, the highest RP yield by immobilized mycelia reached 309.17 CV mL^−1^, a significant increase compared to the original titer. Importantly, this study marks the first report on the successful production of *Monascus* RP in a semi-continuous mode using gamma rays’ mutant strain, offering prospects for commercial production.

## Introduction

Natural pigments include a wide array of bioactive compounds, widely exploited in various industrial applications such as pharmaceuticals, cosmetics, food colourants, dietary supplements, poultry, and aquaculture feed [1]. Recently, natural pigments have led the fast-changing industry compared to synthetic colours. Consequently, demand for natural-produced biopigments will continue to rise. The fungi platform for biopigments production is of great interest as a safe substitute for synthetics. Generally, fungi are efficient biotechnology agents because of their high growth rates [2] and their tolerance to metabolic regulators in large-scale production [3]. Furthermore, in 2000, the EU approved the use of filamentous fungi products, paving the way for a new era of fungi production [4]. In the literature, many microorganisms are known to produce different pigments, but not all these pigments are safe to use. *Monascus* is particularly well-known because it produces various colored edible pigments [5]. Such pigments showed high economic value that attracted worldwide attention as promising coloring agents [6] with easy production, good solubility, high bioactivities, and safety use under specific conditions [7]. Among them, red pigment (RP) is particularly interesting because red is often desired in food colours and it is not easy to obtain natural RPs. In addition, its diverse biological activities include anticancer, antidiabetic, anti-inflammatory, antimicrobial, and antioxidant properties [8, and references therein]. As a result of their functional characteristics, they are promising alternatives to the natural coloring of oriental food products.

The immobilization technique involves the attachment or inclusion of cells into separate carriers, allowing exchanges of substrates, inhibitors, and products, but simultaneously separating catalytic cell biomass from the bulk phase [9, 10]. The application of immobilized technology in the industry has shown much potential in the field of biological fermentation due to its easy operation, repeatability, good stability, maintenance of long-term cell viability, low susceptibility to contamination, and high tolerance [11, 12]. Moreover, gamma radiation was successfully used for the production enhancement of different fungal metabolites including digoxin [13], vinblastine [14], Huperzine A [15], and mycophenolic acid [16].

Until now, very little is available in the literature on the effect of gamma irradiation on pigment production by *Monascus* fungi [17]. Furthermore, information on strategies for the production of *Monascus* RP using the immobilization technique is rare [18]. As such, the constituents of a fermentation medium are known to have a great effect on pigment production by several *Monascus* species [5, 8]. With these objectives in mind, in this paper, we aim to improve RP production by the endophytic strain *Monascus ruber*. Firstly, a strain improvement was applied using gamma irradiation mutagenesis to induce hyperproducer. Second, different entrapping carriers were screened to select the best one for immobilization of the developed mutant strain. Third, optimization of nutritional conditions for maximum RP production. Lastly, the feasibility of RP production in a semi-continuous mode was investigated for the first time.

## Materials and Methods

### Fungal strain

The experimental fungus *Monascus ruber* SRZ112 was isolated from *Origanum majorana* leaves and identified as *Monascus ruber*; GenBank No. MT140350 [19], culture collection No. AUMC14390 of Assiut University Mycological Center, Egypt (https://www.aun.edu.eg/sp_units/en/aumcenter).

### Inoculum preparation and cultivation conditions

Spore suspension of *M. ruber* SRZ112 was prepared from 7-day-old slants and set at 10^6^ spore/mL using a hemocytometer. RP production was conducted using submerged fermentation of 50 ml of PD broth medium in 250 mL Erlenmeyer flasks. 1 mL of the prepared spore suspension was added to the flasks after sterilization and cooling. The inoculated flasks were incubated for 10 days at 25 °C and 120 rpm in darkness.

### Irradiation mutagenesis

The process of irradiation was carried out as previously described at an exposure dose of 1000 Gy of gamma rays, the best dose for the fungal strain [19]. After irradiation, the irradiated suspension was kept in darkness to prevent photoreactivation. After that, the irradiated suspension was diluted by a serial dilution technique, and 100 μL was spread on PDA Petri dishes and then incubated at 25ºC. After 7 days of incubation, the surviving colonies were picked up separately, subcultured, and given a code number.

### Isolation of mutants and screening RP production and stability

The collected mutants of the *M. ruber* SRZ112 were tested for their RP productivities, as described earlier. The highest mutants producing RP (SRZ112—*m06*, SRZ112—*m17*, SRZ112—*m22*, SRZ112—*m41*, SRZ112—*m46*, and SRZ112—*m52*) were tested for their RP production stability for ten successive generations. The highest and most stable mutant SRZ112—*m22* was used to complete the experimental series.

### Genetic characteristics of the parent and mutant

To reveal genomic data of both parent and mutant strands, DNA from both were isolated using the Bead-Beat Micro AX Gravity isolation kit from A&A Biotechnology (Poland, Gdańsk) according to the manufacturer’s instructions. Total isolated DNA quality and quantity were analyzed with TapeStation 4150 from Agilent and with Qubit 4 fluorometer from Thermo Fisher. After quality and quantity control NGS libraries for Whole Genome Sequencing (WGS) were prepared using Oxford Nanopore Technology (ONT) as well as Illumina platform. ONT sequencing was performed with MinION sequencer using SQK-LSK114 chemistry and R10.4.1 flow cells while Illumina MiniSeq sequencing system was used for pair ended (2 × 150 bp) short reads. After sequencing ONT data was basecalled with Dorado 0.5.3 (https://github.com/nanoporetech/dorado) using SUP model, then trimmed with Chopper (https://github.com/wdecoster/chopper) (reads shorter than 300 bp and with phred lower than 12 were discarded as well as 40 bp from the beginning and the end of each read were trimmed). Illumina reads were trimmed with Trimmomatic 0.39 (https://www.ncbi.nlm.nih.gov/pmc/articles/PMC4103590/) with standard parameters (adapters removal, remove leading and tailing bases with low quality, scanning whole sequence with sliding window of four base pairs and cut low-quality fragments). Finally, such prepared data was deposited in the NCBI Sequence Read Archive (SRA) database.

### Cultural and morphological characteristics of the parent and mutant

To study changes in colony morphology, the parent strain and the mutant SRZ112—*m22* were grown on malt extract agar for 10 days at 30 °C. Microscopic observations were taken from fungal growth and stained with cotton blue in lactophenol then examined under the microscope.

### Testing the production of RP by the immobilization technique

Three different entrapment carriers (Na-CMC, sodium alginate, and agar–agar, Merk, Germany) and two different techniques (spore immobilization and mycelia immobilization) were tried to test the effect of immobilized cultures on RP production by *M. ruber* SRZ112—*m22* strain, the highest RP-producing mutant.

Spores and mycelia immobilization using these carriers was adopted according to the method described in detail [9, 12, 20]. The percentages composition of the prepared beads were 2.5%, 3.0%, and 3%, w/v for Na-CMC, sodium alginate, and agar–agar, respectively. The prepared beads of carboxymethyl cellulose sodium salt (Na-CMC), calcium alginate, and agar–agar were washed thrice with deionized water and then 50 beads were transferred to each flask containing 50 mL of medium were used for RP production under the conditions described earlier.

### Optimization of nutritional conditions

#### Selection of the optimum fermentation medium

To select the most proper fermentation broth for RP production, eight different types of media were tested. The names and composition (g L^−1^) of the media are as follows:

1. Potato dextrose: D-glucose 20 and potato infusion 200.
2. Malt extract autolysate: bacteriological peptone 1, glucose 2, malt extract 30, CuSO_4_.5H_2_O 0.005, and ZnSO_4_.7H_2_O 0.01.
3. Sabouraud’s-glucose: glucose 20, bactopeptone 10, KH_2_PO_4_ 1.0, and MgSO_4_.7H_2_O 1.0.
4. Modified Lin’s: glucose 30.0, monosodium glutamate (MSG) 1.5, KH_2_PO_4_ 2.5, MgSO_4_.7H_2_O 1, and FeSO_4_.7H_2_O 0.014 [21].
5. Czapek-Dox’s: NaNO3 3, sucrose 30, MgSO_4_.7H_2_O 0.5, KH_2_PO_4_ 0.5, KCl 0.5, and FeSO_4_.7H_2_O 0.013.
6. Modified medium A: MSG 10, glucose 10, MgSO_4_.7H_2_O 0.5, K_2_HPO_4_ 5, KH_2_PO_4_ 5, ZnSO_4_.7H_2_O 0.01, CaCl_2_ 0.1, FeSO_4_.7H_2_O 0.01, and MnSO_4_.4H_2_O 0.03 [22].
7. Yeast-sucrose: yeast extract 20, sucrose 50, CuSO_4_.7H_2_O 0.005, MgSO_4_.7H_2_O 0.05, and ZnSO_4_.7H2O 0.01.
8. Optimized medium: potato infusion 200, glycerol 25, peptone 51, D-glucose 20, and NaCl 12.5 [23].

#### Response surface methodology (RSM) optimization of medium components

Modified Lin’s medium components of glucose, MSG, KH_2_PO_4_, MgSO_4_.7H_2_O, and FeSO_4_.7H_2_O were optimized by RSM using Box–Behnken design [24] using the software Design–Expert version 8.0.7.1. Table 4 presents the trials for the five media components and their actual and coded levels. The broth media were separately inoculated with 50 beads of immobilized spores or mycelia, as previously described.The data obtained on RP production (for both spore-immobilized and mycelia-immobilized cultures) from the RSM program was analyzed by ANOVA (analysis of variance). The statistical significance was determined by Fisher's F test, and the model's explanation of the proportion of variance is given by coefficients of determination, R^2^ value. The 2D contour and 3D response surface plots were generated based on response analysis to explain the interaction between the five factors.

##### Validation of the models

 Additional independent experiments were conducted to validate the performance of the optimum design levels to maximize RP production. The actual yield of the RP and the yield predicted by the model were compared.

#### Production of RP using cell recycle batch fermentation

Alginate beads were separated under aseptic conditions from the fermentation broth at the end of each cycle. The whole broth at the end of the cycle was filtered (sterile Buchner funnel) and the beads were washed thrice with deionized sterile water. The collected beads from the previous cycle were used in the next cycle after adding fresh medium then the flasks were incubated under the conditions described earlier. The described sequence of cultivation, separation of the beads, and their reuse was repeated for eight different cycles.

### Analytical methods

#### Determination of dry biomass

In the case of free submerged fermentation, biomass was collected by filtration at the end of incubation and then weighed until a constant weight at 50 °C. Meanwhile, in the case of immobilized cultures, beads with mycelia were collected by filtration then re-suspended in pH 7.5 phosphate buffer and placed for 2 h on a rotary (200 rpm) shaker. The released mycelia were collected by filtration then washed, and finally dried (50 °C).

#### Estimation of RP yield

RP production was estimated by a JENWAY-305 (UK) spectrophotometer at a wavelength set at 500 nm in terms of absorbance units per mL of the fermentation filtrate according to a previously reported method [25], taking into account the dilution factor of the sample. The RP yield was given as units of color value per mL of fermentation filtrate (CV mL^−1^) multiplied by the dilution factor [26].

### Statistical analysis

The calculated mean is for measurements made in triplicate from two experiments. The statistical significance was analysed by the analysis of variance (One-Way ANOVA) and least significant difference (at 0.05 level) tests using SPSS software version 22 (IBM Corp).

## Results and discussion

### Developing a stable mutant for enhanced RP production

**Fig. 1:**
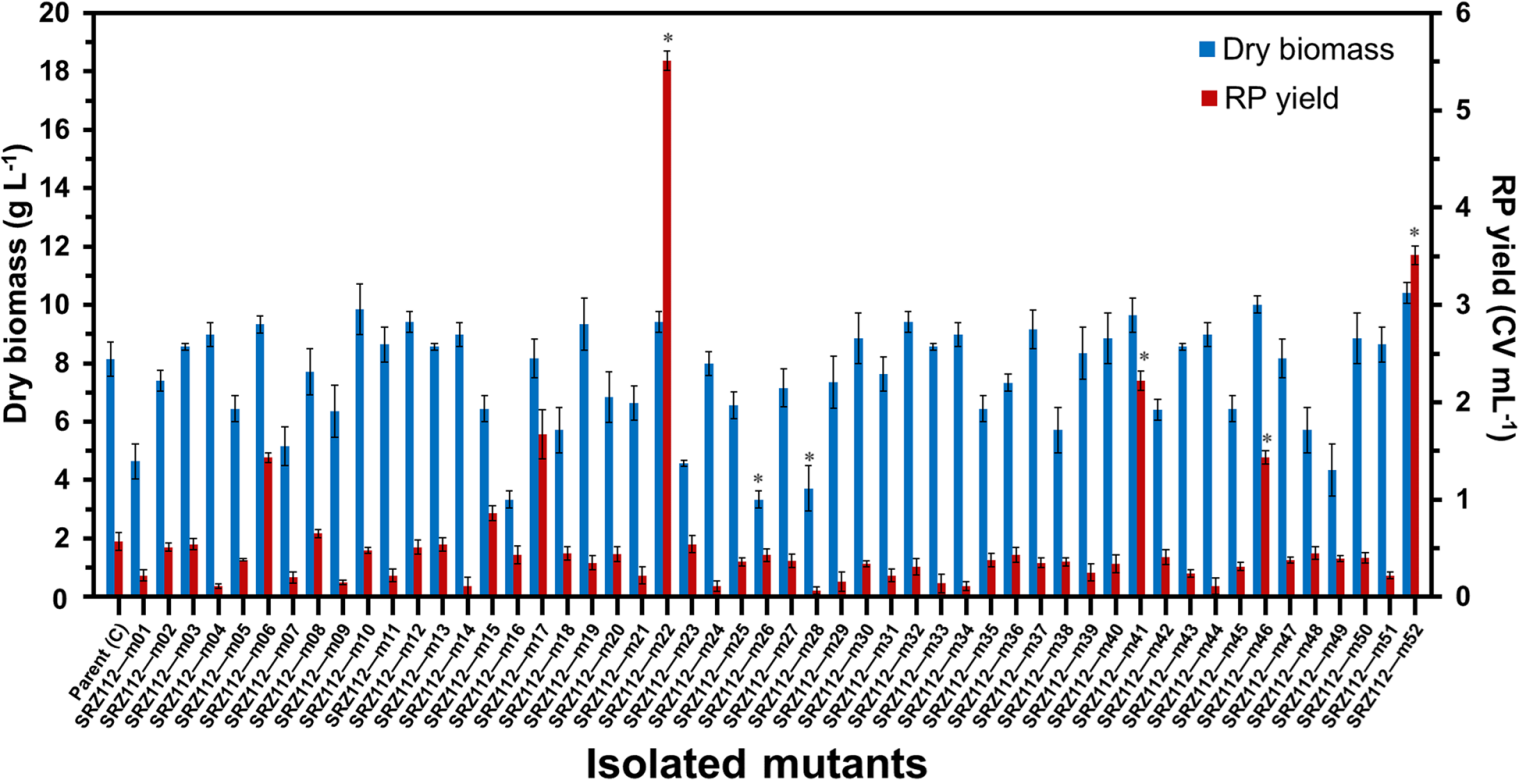
Dry biomass (g L^−1^) and RP yield (CV mL.^−1^) by *Monascus ruber* SRZ112 parent and the isolated mutants. Cultures were grown in 50 mL PD medium (pH 6.0) inoculated with 1 mL inoculum size of 7-day-old culture and incubated at 120 rpm and 25ºC for 10 days. The calculated mean is for triplicate measurements from two independent experiments. The means with * are significantly different from the control (LSD test, *P* ≤ 0.05)

The survivor colonies, after exposure to gamma rays, were collected and their RP production was evaluated. Remarkable variations in the RP yields from the collected mutants were observed where significant (*P* ≤ *0.05)* differences in the recorded values of CV mL^−1^ were obtained compared with the control (Fig. 1). As a general observation, the application of gamma rays (1.00 kGy) induced positive mutants where the RP yield increased as well negative mutants where the RP yield was significantly decreased, as shown in Fig. 1. The RP yield range of the separated mutants was from 0.11 CV mL^−1^ to 5.51 CV mL^−1^. Figure 1 also shows that the mutants SRZ112—*m06*, SRZ112—*m17*, SRZ112—*m22*, SRZ112—*m41*, SRZ112—*m46*, and SRZ112—*m52* were the highest producers of RP compared to the control (parent). Therefore, they were followed by ten successive generations for their stability in RP production. As shown in Table 1, the mutant SRZ112—*m22* had RP production stability across the ten generations where no significant (*P* ≤ *0.05)* differences were recorded. However, RP yields from the other mutants SRZ112—*m06*, SRZ112—*m17*, SRZ112—*m41*, SRZ112—*m46*, and SRZ112—*m52* were unstable. Table 1 also shows that the achieved RP yield from *M. ruber* SRZ112—*m22* cultures was 9.67 times higher than the RP yield from cultures of the parent. Generally, exposing microbial cells to mutagenic agents is a classical and simple method for microbial strain improvement [27, and references therein] where mutagens (physical, chemical, and biological) can induce mutations in microbial genes [18]. Such mutations can artificially regulate metabolic pathways and intensify the titers of the desired product [28, 29]. Consequently, exposure of *M. ruber* SRZ112 spores to gamma rays can have a stimulatory effect on RP production and up-regulate it in the mutant *M. ruber* SRZ112—*m22*. A previous report used plasma rays at room temperature to induce a mutant strain of *M*. *purpureus* and the resultant mutant (designed as M630) showed high pigment production [30]. Our results (Table 1) also indicated the negative effect of gamma rays on the cellular growth of *M. ruber* SRZ112. Following our results, the same observation on the reducing effect of gamma rays on the growth of *Aspergillus fumigatus* and *Alternaria tenuissima* [31], *Aspergillus sydowii*, *Aspergillus flavus* [32], and *Epicoccum nigrum* [33] was reported. In the literature, gamma radiation at certain exposure doses was highly recommended in several reports to improve different fungi (*Alternaria alternata, Penicillium roqueforti, Epicoccum nigrum, Alternaria brassicae*) overexpressing several industrially significant metabolites [13–16].

**Table 1:** Dry biomass (g L^-1^) and RP production (CV mL^-1^ culture filtrate) of the isolated mutant strains grown for ten successive generations

Generation	SRZ112–*m06*		SRZ112–*m17*		SRZ112–*m22*		SRZ112–*m41*		SRZ112–*m46*		SRZ112–*m52*	
	Dry biomass (g L^−1^)	RP yield (CV mL^−1^)	Dry biomass (g L^−1^)	RP yield (CV mL^−1^)	Dry biomass (g L^−1^)	RP yield (CV mL^−1^)	Dry biomass (g L^−1^)	RP yield (CV mL^−1^)	Dry biomass (g L^−1^)	RP yield (CV mL^−1^)	Dry biomass (g L^−1^)	RP yield (CV mL^−1^)
First	9.37 ± 0.12^a^	1.52 ± 0.98^a^	8.18 ± 0.18^a^	1.71 ± 0.11^c^	9.68 ± 0.56^b^	5.32 ± 0.14^a^	9.36 ± 0.32^a^	2.32 ± 1.09^a^	10.22 ± 0.82^a^	1.48 ± 1.08^a^	9.76 ± 0.82^a^	3.30 ± 0.83^a^
Second	9.11 ± 0.19^a^	1.03 ± 0.19^a^	7.96 ± 0.43^a^	1.33 ± 0.05^ cd^	9.49 ± 0.12^a^	5.18 ± 0.09^a^	9.41 ± 0.51^a^	0.87 ± 1.09^b^	9.98 ± 0.39^a^	0.98 ± 0.07^ab^	9.62 ± 0.71^a^	2.41 ± 0.41^b^
Third	9.65 ± 0.48^a^	0.95 ± 0.03^ab^	8.01 ± 0.18^a^	1.04 ± 0.04^ cd^	9.37 ± 0.75^a^	5.42 ± 0.11^a^	9.52 ± 0.34^a^	0.92 ± 1.09^b^	10.10 ± 0.91^a^	0.92 ± 0.07^ab^	8.99 ± 0.44^a^	2.56 ± 0.91^b^
Fourth	9.56 ± 0.21^a^	0.89 ± 0.09^ab^	7.98 ± 0.21^a^	2.47 ± 0.16^b^	9.68 ± 0.70^a^	5.51 ± 0.09^a^	9.38 ± 0.47^a^	0.79 ± 1.09^b^	10.02 ± 0.82^a^	1.01 ± 0.21^ab^	8.82 ± 0.82^a^	3.78 ± 0.76^a^
Fifth	9.44 ± 0.72^a^	0.93 ± 0.11^ab^	8.31 ± 0.28^a^	1.01 ± 0.04^d^	9.24 ± 0.85^a^	5.37 ± 0.08^a^	9.49 ± 0.71^a^	0.56 ± 1.09^b^	10.11 ± 0.75^a^	0.85 ± 0.55^b^	9.39 ± 0.39^a^	1.22 ± 0.52^c^
Sixth	9.76 ± 0.37^a^	0.75 ± 0.03^ab^	8.21 ± 0.16^a^	3.00 ± 0.17^a^	9.71 ± 0.71^a^	5.76 ± 0.10^a^	9.30 ± 0.41^a^	0.59 ± 1.09^b^	9.87 ± 0.92^a^	0.42 ± 0.01^c^	9.46 ± 0.76^a^	1.00 ± 0.11^c^
Seventh	9.92 ± 0.63^a^	0.53 ± 0.10^b^	8.33 ± 0.36^a^	1.21 ± 0.09^ cd^	9.51 ± 0.32^a^	5.59 ± 0.14^a^	9.43 ± 0.67^a^	0.61 ± 1.09^b^	10.05 ± 0.78^a^	0.57 ± 0.01^c^	9.59 ± 0.31^a^	1.21 ± 0.18^c^
Eighth	9.52 ± 0.48^a^	0.57 ± 0.04^b^	8.87 ± 0.41^a^	0.89 ± 0.03^d^	9.63 ± 0.87^a^	5.38 ± 0.09^a^	8.99 ± 0.98^a^	0.58 ± 1.09^b^	10.21 ± 0.18^a^	0.56 ± 0.04^c^	9.81 ± 0.19^a^	0.56 ± 0.08^d^
Ninth	9.31 ± 0.11^a^	0.55 ± 0.03^b^	8.00 ± 0.21^a^	0.76 ± 0.01^d^	9.77 ± 0.89^a^	5.41 ± 0.08^a^	9.81 ± 0.31^a^	0.71 ± 1.09^b^	10.31 ± 0.76^a^	0.57 ± 0.02^c^	9.77 ± 0.45^a^	0.58 ± 0.01^d^
Tenth	9.56 ± 0.37^a^	0.53 ± 0.07^b^	8.34 ± 0.36^a^	0.84 ± 0.09^d^	9.65 ± 0.55^a^	5.41 ± 0.11^a^	9.55 ± 0.71^a^	0.59 ± 1.09^b^	10.19 ± 0.82^a^	0.55 ± 0.03^c^	9.08 ± 0.39^a^	0.57 ± 0.06^d^

Cultures were grown in 50 mL PD medium (pH 6.0) inoculated with 1 mL inoculum size of 7-day-old culture and incubated at 120 rpm and 25ºC for 10 days. The calculated mean is for triplicate measurements from two independent experiments. The means with different superscripts in the same column are considered statistically different (LSD test, P ≤ 0.05)

**Fig. 2:**
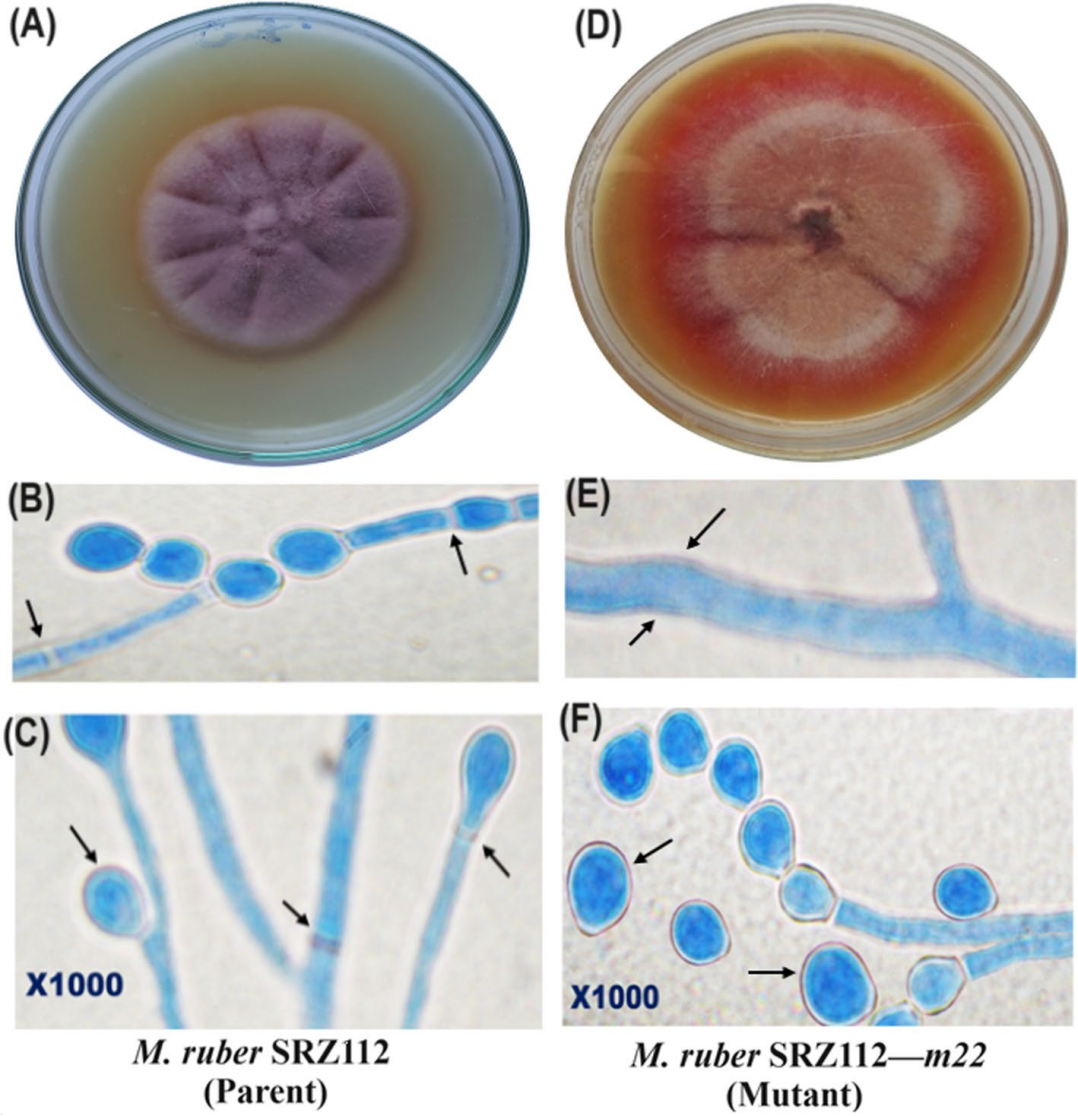
Colony phenotype and microscopic morphology of the parent strain* M. ruber* SRZ112 (**A**) and the developed mutant *M. ruber* SRZ112—*m22* (**B**). Colony growth was observed on Czapek–Yeast autolysate agar after incubation for 10 days at 25 °C. The appearance of both strains was studied under the light microscope after staining with lactophenol cotton blue

Genomic data obtained for parent strain was: i) 1.66 Gbp in total with N50 equal 5121 bp and 19.7 median read quality for ONT sequencing, ii) 852.5 Mbp with 2 × 2 902 727 reads for Illumina pair-end sequencing. Analogical data for the mutant strain was: i) 1.14 Gbp in total with N50 equal 4,238 bp and 19.6 median read quality for ONT data, ii) 2.3 Gbp 2 × 8 173 861 reads for Illumina pair-end sequencing. Both WGS datasets were deposited in the SRA database under PRJNA1091857 and PRJNA1091829 for ONT and Illumina libraries, respectively. Regarding the effect of gamma rays on colony morphology and microscopic characteristics of the mutant developed, Fig. 2 indicates the differences between the parent strain (Fig. 2A) and the mutant strain SRZ112—*m22* (Fig. 2D) on the surface and colony diameter of the mutant. Moreover, colonies of the parent strain exhibited light pigmentation; however, the mutant colonies had a deeper red color. According to previous reports [17, 34], the colony diameter of the irradiated fungi was different from the parent. Figure 2 further shows that significant differences in conidia formation were observed. The numbers and size of the conidia for the mutant (Fig. 2F) were noticeably higher than those of the parent (Fig. 2C). In addition, the conidia of the mutant strain had a thicker wall than the conidia of the parent. The hyphae of the parent strain were hyaline, with thin walls and septa (Fig. 2B). However, the hyphae of the mutant strain were tangled, wider, and thicker (Fig. 2E). Similarly, previous studies reported that changes in hyphae diameter were very extensive, as the diameter of the hyphae in the irradiated fungi was greater than that of the control [17, 34].

### Production of RP by immobilization technique

Here, RP production by *M. ruber* SRZ112—*m22* immobilized spores and mycelia using three entrapment carriers were studied under submerged fermentation. The obtained data (Table 2), generally indicated that the type of immobilized mass (spores or mycelia) and the applied carrier had a significant effect on both the growth and RP yield. In accordance with our results, previous reports [31, 35, 36] attributed the superiority of an entrapment carrier over other carriers to the nature of and the immobilized organism and the used carrier itself. Our results also indicated that the RP yield of the immobilized mycelia of *M. ruber* SRZ112—*m22* was higher than the RP yield of the immobilized spores. Previous reports recorded the same observations with different fungi and metabolites [9, 31, 35].

**Table 2:** Dry biomass (g L^-1^) and RP production (CV mL^-1^ culture filtrate) of *M. ruber *SRZ112–*m22* immobilized by different spore and mycelia entrapping carriers

Entrapment carrier	Immobilized spores		Immobilized mycelia	
	Dry biomass (g L^-1^)	RP yield (CV mL^-1^)	Dry biomass (g L^-1^)	RP yield (CV mL^-1^)
Free cultures (C)	9.52±0.47^a^	5.44±0.09^c^	12.43±0.86^a^	5.87±0.13^c^
Calcium alginate	5.87±0.08^ab^	7.24±0.12^a^	7.67±0.05^b^	8.29±0.38^a^
Agar-agar	4.96±0.11^b^	6.11±0.57^b^	6.51±0.08^b^	7.01±0.59^b^
Na-CMC	4.07±0.13^b^	6.45±0.10^b^	6.87±0.12^b^	7.21±0.42^b^

Cultures were grown in 50 mL PD medium (pH 6.0) inoculated with 50 beads and incubated at 120 rpm and 25ºC for 10 days. The calculated mean is for triplicate measurements from two independent experiments. The means with different superscripts in the same column are considered statistically different (LSD test, *P* ≤ 0.05)

The data presented in Table 2 showed that alginate was the best carrier for the highest RP production by both spores and mycelia of *M. ruber* SRZ112—*m22*. The achieved RP yields using this carrier were 7.24 ± 0.12 and 8.29 ± 0.38 for spores- and mycelia-immobilized cultures, respectively. Interestingly, spores- and mycelia-immobilized cultures produced 1.33 and 1.41 times higher than the free control cultures even with lower cell growth compared to free cultures. Our results (Table 2) also showed that satisfactory RP yields were achieved by using Na-CMC followed by agar–agar. In concurrence with these results, several reports used alginate as the immobilization carrier for the production of different metabolites [31, 35, 36]. Alginates, amongst other carriers, are the most promising immobilization matrix because they are cheap support materials with mechanical stability [37]. It is a family of the binary copolymer of (1 → 4)-linked β-D-mannuronic acid and α-L-guluronic acid [38]. Alginates are mechanically strong with low shrinkage and high porosity during gel formation due to their high guluronic acid content [39]. Furthermore, the simple preparations of gel beads, the widely varying composition, and sequential structure, their use in the whole-cell immobilization technology is highly recommended [40]. On the contrary, the superiority of CMC-immobilized cells for the production of polysaccharides over others was reported [36]. Due to its harmlessness, biocompatibility, low-cost, high-water absorption, and biodegradability, CMC has been widely applied in several immobilization fermentations [9, 31].

### Selection of the optimum fermentation medium for maximum RP production

Here, eight different fermentation media were tested (Table 3) as a starting step in designing and optimizing the medium for maximum RP production by* M. ruber* SRZ112—*m22*. The data in Table 3 indicated that the maximum RP yield (10.51 ± 1.01 CV mL^−1^ for immobilized spores and 12.54 ± 1.08 CV mL^−1^ for immobilized mycelia) was achieved using modified Lin’s medium broth. Moreover, modified medium A (9.46 ± 0.81 CV mL^−1^ for immobilized spores and 10.41 ± 0.91 CV mL^−1^ for immobilized mycelia) followed by Optimized medium (8.59 ± 0.71 CV mL^−1^ for immobilized spores and 10.09 ± 0.84 CV mL^−1^ for immobilized mycelia) were good media for RP production. However, the lowest RP yields were recorded using Czapek-Dox's broth. Several reports concluded that the constituents of the fermentation broth have a great influence on pigment production by different *Monascus* species [5, 18]. For example, the same broth medium was used for maximum RP production by *M. purpureus* ATCC16365 [21]. Moreover, modified medium A for pigment production by *M. sanguineus* [23] and optimized medium for the production of RP by *Monascus ruber* [22]. Accordingly, such superiority of the Modified Lin broth could be attributed to the presence of specific nutrients such as carbon, nitrogen, and phosphorus sources, as well as other micronutrients that play a positive role in RP production. In general, no single medium is the best for all types of fungi, thus specific nutrients and cultivation conditions are intrinsically related to the nature of the fungus [41].

**Table 3:** Effect of different fermentation media on dry biomass (g L^-1^) and RP production (CV mL^-1^ culture filtrate) of immobilized spores and mycelia of *M. ruber *SRZ112–*m22* in calcium alginate beads

Broth medium	Immobilized spores		Immobilized mycelia	
	Dry biomass (g L^-1^)	RP yield (CV mL^-1^)	Dry biomass (g L^-1^)	RP yield (CV mL^-1^)
1. Potato dextrose (Control)	5.87±0.21^c^	7.24±0.58^ab^	7.45±0.35^ab^	8.44±0.95^bc^
2. Malt extract autolysate	6.76±0.73^bc^	5.71±0.54^b^	8.29±0.43^ab^	9.32±0.81^bc^
3. Sabouraud’s-glucose	7.66±0.61^bc^	5.33±0.66^b^	8.44±0.29^ab^	4.08±0.32^cd^
4. Modified Lin’s medium	8.43±0.98^a^	10.51±1.01^a^	9.78±0.16^a^	12.54±1.08^a^
5. Czapek-Dox’s	4.61±0.39^cd^	1.21±0.92^cd^	5.81±0.54^b^	2.11±0.32^d^
6. Modified medium A	8.43±0.98^a^	9.46±0.81^a^	9.77±0.48^a^	10.41±0.91^a^
7. Yeast-sucrose	6.91±0.80^bc^	5.96±0.81^b^	6.01±0.39^b^	4.78±0.08^cd^
8. Optimized medium	8.43±0.98^a^	8.59±0.71^ab^	9.37±0.28^a^	10.09±0.84^a^

The initial pH of all tested media was adjusted to 6.0 using 1N NaOH and HCl. Cultures were grown in a 50 mL medium inoculated with 50 calcium alginate beads and incubated at 120 rpm and 25ºC for 10 days. The calculated mean is for triplicate measurements from two independent experiments. The means with different superscripts in the same column are considered statistically different (LSD test, *P* ≤ 0.05)

### Response surface methodology (RSM) optimization of medium components

Modified Lin’s medium components of glucose, MSG, KH_2_PO_4_, MgSO_4_.7H_2_O, and FeSO_4_.7H_2_O were optimized by RSM and Table 4 presents the two Box–Behnken designs. The results obtained for both immobilized spores and mycelia were analysed and the following equations were obtained:

$$RP\;yield\;(immobilized\;spores)=+63.68+6.48A+12.16B+1.86C+1.42D+3.53E+3.02AB+7.28AC+2.13AD+1.58AE+0.2825BC-7.87BD+5.11BE-2.41CD+1.96CE+2.49DE-22.79A^2-15.06B^2-21.44C^2-23.45D^2-30.65E^2$$ 

$$RP\;yield\;(immobilized\;mycelia)=+83.68+6.79A+12.97B+0.9894C+1.47D+4.82E-1.48AB+14.28AC+2.63AD-1.17AE+1.78BC-10.37BD+3.86BE+1.84CD+2.21CE+1.06DE-23.34A^2-13.94B^2-24.07C^2-19.97D^2-34.68E^2$$ 

where A, B, C, D, and E are the symbols of glucose, MSG, KH_2_PO_4_, MgSO_4_⋅7H_2_O, and FeSO_4_⋅7H_2_O, respectively.

**Table 4:** BB experimental design matrix representing the response of RP production (CV mL^-1^ culture filtrate) by immobilized spores and mycelia of *M. ruber* SRZ112–*m22* in calcium alginate beads

Run	Factor A: Glucose (g L^-1^)		Factor B: MSG (g L^-1^)		Factor C: KH_2_PO_4_ (g L^-1^)		Factor D: MgSO_4_⋅7H_2_O (g L^-1^)		Factor E: FeSO_4_⋅7H_2_O (g L^-1^)		RP yield (CV mL^-1^)			
	Actual	Coded	Actual	Coded	Actual	Coded	Actual	Coded	Actual	Coded	Immobilized spores		Immobilized mycelia	
											Actual	Predicted	Actual	Predicted
1	5	(-1)	5	(-1)	1.5	(0)	1	(0)	0.55	(0)	10.47±0.98	10.21	26.23±1.43	25.16
2	35	(1)	5	(-1)	1.5	(0)	1	(0)	0.55	(0)	14.53±1.02	17.13	41.65±3.51	41.70
3	5	(-1)	35	(1)	1.5	(0)	1	(0)	0.55	(0)	30.82±3.11	28.49	55.38±6.12	54.06
4	35	(1)	35	(1)	1.5	(0)	1	(0)	0.55	(0)	46.97±2.87	47.50	64.89±5.26	64.69
5	20	(0)	20	(0)	0.5	(-1)	0.5	(-1)	0.55	(0)	13.77±0.98	13.09	39.76±3.44	39.02
6	20	(0)	20	(0)	2.5	(1)	0.5	(-1)	0.55	(0)	21.63±2.41	21.65	38.94±3.71	37.32
7	20	(0)	20	(0)	0.5	(-1)	1.5	(1)	0.55	(0)	21.67±1.66	20.77	38.56±2.22	38.28
8	20	(0)	20	(0)	2.5	(1)	1.5	(1)	0.55	(0)	19.88±2.09	19.67	45.09±1.09	43.94
9	20	(0)	5	(-1)	1.5	(0)	1	(0)	0.1	(-1)	8.67±0.62	7.39	21.93±2.11	21.12
10	20	(0)	35	(1)	1.5	(0)	1	(0)	0.1	(-1)	17.55±1.11	21.49	39.91±1.62	39.35
11	20	(0)	5	(-1)	1.5	(0)	1	(0)	1	(1)	6.65±0.32	4.22	22.39±3.22	23.05
12	20	(0)	35	(1)	1.5	(0)	1	(0)	1	(1)	35.96±4.15	38.76	55.80±5.31	56.71
13	5	(-1)	20	(0)	0.5	(-1)	1	(0)	0.55	(0)	17.66±2.33	18.39	42.45±4.03	42.77
14	35	(1)	20	(0)	0.5	(-1)	1	(0)	0.55	(0)	17.16±1.35	16.80	28.36±2.47	27.80
15	5	(-1)	20	(0)	2.5	(1)	1	(0)	0.55	(0)	7.16±1.65	7.56	16.75±1.71	16.19
16	35	(1)	20	(0)	2.5	(1)	1	(0)	0.55	(0)	35.77±1.43	35.08	59.77±3.90	58.34
17	20	(0)	20	(0)	1.5	(0)	0.5	(-1)	0.1	(-1)	6.16±0.41	7.12	24.97±2.31	23.80
18	20	(0)	20	(0)	1.5	(0)	1.5	(1)	0.1	(-1)	4.47±0.25	4.99	23.70±1.99	24.62
19	20	(0)	20	(0)	1.5	(0)	0.5	(-1)	1	(1)	8.53±0.99	9.19	32.90±2.42	31.32
20	20	(0)	20	(0)	1.5	(0)	1.5	(1)	1	(1)	16.81±1.52	17.03	35.86±2.89	36.38
21	20	(0)	5	(-1)	0.5	(-1)	1	(0)	0.55	(0)	11.18±0.91	13.44	34.06±2.51	33.49
22	20	(0)	35	(1)	0.5	(-1)	1	(0)	0.55	(0)	37.87±2.56	37.20	55.70±5.01	55.87
23	20	(0)	5	(-1)	2.5	(1)	1	(0)	0.55	(0)	15.05±1.41	16.60	30.35±1.33	31.90
24	20	(0)	35	(1)	2.5	(1)	1	(0)	0.55	(0)	42.87±5.22	41.49	59.11±2.76	61.42
25	5	(-1)	20	(0)	1.5	(0)	0.5	(-1)	0.55	(0)	9.74±2.10	11.67	33.98±1.45	34.74
26	35	(1)	20	(0)	1.5	(0)	0.5	(-1)	0.55	(0)	19.29±1.62	20.38	41.19±3.71	43.07
27	5	(-1)	20	(0)	1.5	(0)	1.5	(1)	0.55	(0)	8.98±1.03	10.26	31.20±1.82	32.42
28	35	(1)	20	(0)	1.5	(0)	1.5	(1)	0.55	(0)	27.04±2.66	27.48	48.92±3.29	51.27
29	20	(0)	20	(0)	0.5	(-1)	1	(0)	0.1	(-1)	9.96±0.43	8.16	20.74±1.01	21.33
30	20	(0)	20	(0)	2.5	(1)	1	(0)	0.1	(-1)	9.44±0.91	7.97	18.68±1.59	18.90
31	20	(0)	20	(0)	0.5	(-1)	1	(0)	1	(1)	9.86±1.43	11.30	25.51±3.22	26.56
32	20	(0)	20	(0)	2.5	(1)	1	(0)	1	(1)	17.16±2.02	18.94	32.27±3.19	32.95
33	5	(-1)	20	(0)	1.5	(0)	1	(0)	0.1	(-1)	1.79±0.21	1.82	11.80±0.95	12.89
34	35	(1)	20	(0)	1.5	(0)	1	(0)	0.1	(-1)	12.51±3.11	11.61	29.09±1.43	28.81
35	5	(-1)	20	(0)	1.5	(0)	1	(0)	1	(1)	7.47±1.51	5.70	25.30±2.98	24.86
36	35	(1)	20	(0)	1.5	(0)	1	(0)	1	(1)	24.53±3.66	21.83	37.94±4.19	36.12
37	20	(0)	5	(-1)	1.5	(0)	0.5	(-1)	0.55	(0)	5.59±1.00	3.71	23.34±2.62	24.95
38	20	(0)	35	(1)	1.5	(0)	0.5	(-1)	0.55	(0)	45.88±6.21	43.78	70.78±10.33	71.64
39	20	(0)	5	(-1)	1.5	(0)	1.5	(1)	0.55	(0)	22.87±2.44	22.31	50.06±9.45	48.64
40	20	(0)	35	(1)	1.5	(0)	1.5	(1)	0.55	(0)	31.67±4.11	30.89	56.01±6.10	53.84
41	20	(0)	20	(0)	1.5	(0)	1	(0)	0.55	(0)	64.41±3.76	63.68	84.41±9.42	83.68
42	20	(0)	20	(0)	1.5	(0)	1	(0)	0.55	(0)	63.82±4.21	63.68	83.82±10.77	83.68
43	20	(0)	20	(0)	1.5	(0)	1	(0)	0.55	(0)	63.55±4.61	63.68	83.55±8.49	83.68
44	20	(0)	20	(0)	1.5	(0)	1	(0)	0.55	(0)	64.92±3.09	63.68	84.92±10.41	83.68
45	20	(0)	20	(0)	1.5	(0)	1	(0)	0.55	(0)	61.55±3.22	63.68	81.55±7.29	83.68
46	20	(0)	20	(0)	1.5	(0)	1	(0)	0.55	(0)	63.85±2.61	63.68	83.85±10.33	83.68

Cultures were grown in 50 mL Modified Lin’s medium (pH 6.0) inoculated with 50 calcium alginate beads and incubated at 120 rpm and 25ºC for 10 days. The calculated mean is for triplicate measurements from two independent experiments

**Fig. 3:**
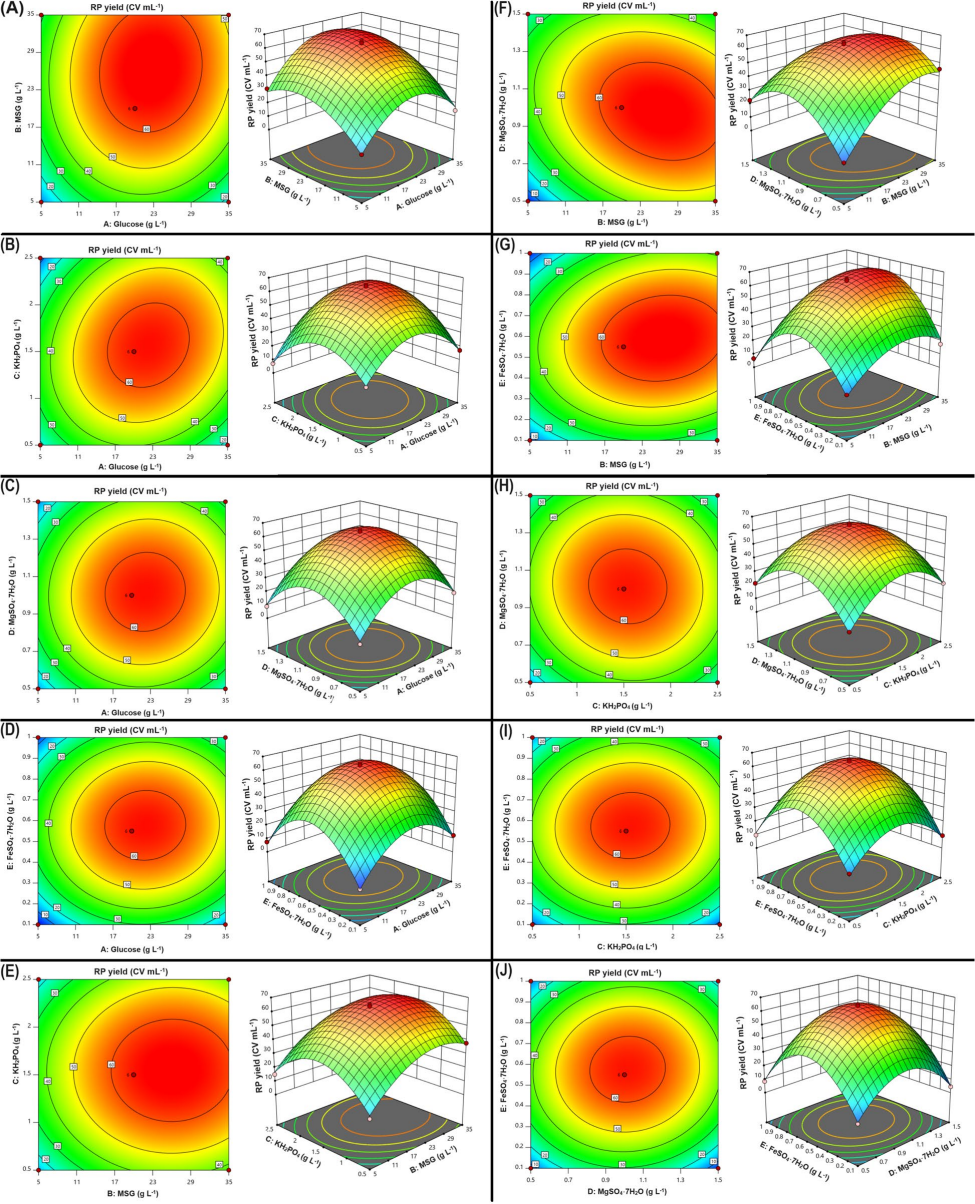
2D contour and 3D surface plots showing the effect of medium components on RP yield by immobilized spores of *M. ruber* SRZ112—*m22* in alginate beads

**Fig. 4:**
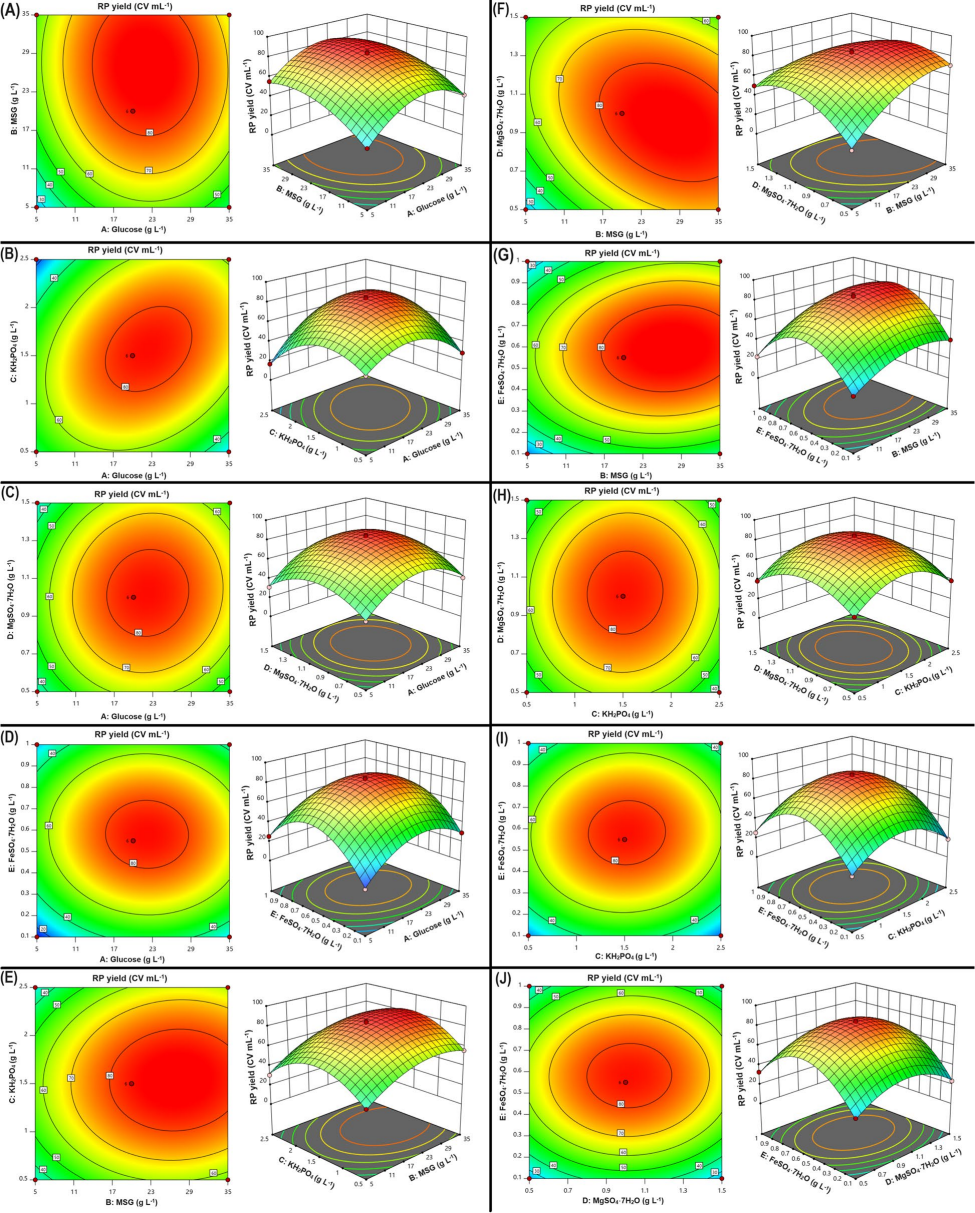
2D contour and 3D surface plots showing the effect of medium components on RP yield by immobilized mycelia of *M. ruber* SRZ112—*m22* in alginate beads

The significance of the second-order polynomial for RP yields from cultures of immobilized spores and mycelia was determined by an analysis of variance as shown in Table 5. The two model terms were significant; a model with a very low probability value of less than 0.0001 confirms that the model significantly fit to the experimental data. Both values of the coefficients of determination (R^2^) of immobilized spores and mycelia models were 0.9937 and 0.9968 indicating the model adequately represented the relationship between the medium components (tested factors) and RP yield (response). Table 5. further shows the coefficient of variation (C.V.) of the two models confirming the degree of precision with which the two models were carried out. Moreover, the lack of fits of both immobilized spores and mycelia models are insignificant (Table 5). To detect the optimal levels of glucose, MSG, KH_2_PO_4_, MgSO_4_.7H_2_O, and FeSO_4_.7H_2_O, graphical representations of the regression Eqs. (2D contour and 3D response surface plots, Figs. 3 and 4) were generated. The study of Figs. 3 and 4 indicated that maximum RP yields (65.651 CV mL^−1^ for spores and 86.140 for mycelia CV mL^−1^) were attained when the concentration of glucose, glucose, MSG, KH_2_PO_4_, MgSO_4_⋅7H_2_O, FeSO_4_⋅7H_2_O were 23.54, 28.77, 1.69, 1.047, and 0.545 g L^−1^, respectively. Similarly, RP production by *M. purpureus* MTCC 369 [42] and *M. purpureus* M183 [43] was optimized confirming that RSM may be successfully applied for the optimization of medium components.

**Table 5:** Analysis of variance (ANOVA) for BB experimental design matrix representing the response of RP production (CV mL^-1^ culture filtrate) by immobilized spores and mycelia of *M. ruber* SRZ112–*m22* in calcium alginate beads

Source	Immobilized spores					Immobilized mycelia				
	SS	d.f.	MS	*F*-value	Prob > *F*	SS	d.f.	MS	*F*-value	Prob > *F*
Model	16236.59	20	811.83	199.50	< 0.0001	19227.86	20	961.39	399.43	< 0.0001
A	672.24	1	672.24	165.20	< 0.0001	738.61	1	738.61	306.87	< 0.0001
B	2366.34	1	2366.34	581.52	< 0.0001	2693.09	1	2693.09	1118.90	< 0.0001
C	55.61	1	55.61	13.67	0.0011	15.66	1	15.66	6.51	0.0172
D	32.49	1	32.49	7.98	0.0091	34.62	1	34.62	14.38	0.0008
E	198.95	1	198.95	48.89	< 0.0001	372.07	1	372.07	154.58	< 0.0001
AB	36.54	1	36.54	8.98	0.0061	8.73	1	8.73	3.63	0.0684
AC	211.85	1	211.85	52.06	< 0.0001	815.39	1	815.39	338.77	< 0.0001
AD	18.11	1	18.11	4.45	0.0451	27.62	1	27.62	11.47	0.0023
AE	10.05	1	10.05	2.47	0.1286	5.43	1	5.43	2.26	0.1457
BC	0.3192	1	0.3192	0.0784	0.7817	12.71	1	12.71	5.28	0.0302
BD	247.91	1	247.91	60.92	< 0.0001	430.36	1	430.36	178.80	< 0.0001
BE	104.35	1	104.35	25.64	< 0.0001	59.52	1	59.52	24.73	< 0.0001
CD	23.28	1	23.28	5.72	0.0246	13.51	1	13.51	5.61	0.0259
CE	15.29	1	15.29	3.76	0.0640	19.45	1	19.45	8.08	0.0088
DE	24.85	1	24.85	6.11	0.0206	4.48	1	4.48	1.86	0.1846
A^2^	4532.48	1	4532.48	1113.84	< 0.0001	4752.73	1	4752.73	1974.62	< 0.0001
B^2^	1980.25	1	1980.25	486.64	< 0.0001	1696.85	1	1696.85	704.99	< 0.0001
C^2^	4011.39	1	4011.39	985.78	< 0.0001	5056.13	1	5056.13	2100.67	< 0.0001
D^2^	4798.47	1	4798.47	1179.21	< 0.0001	3481.67	1	3481.67	1446.53	< 0.0001
E^2^	8200.38	1	8200.38	2015.21	< 0.0001	10495.42	1	10495.42	4360.53	< 0.0001
Lack of Fit	95.06	20	4.75	3.56	0.0818	53.50	20	2.67	2.00	0.2266
	S.D.	2.01	R^2^		0.9937	S.D.	1.55	R^2^		0.9968
	Mean	24.24	Adjusted R^2^		0.9886	Mean	43.33	Adjusted R^2^		0.9943
	C.V. %	8.32	Predicted R^2^		0.9761	C.V. %	3.58	Predicted R^2^		0.9884
	PRESS	389.84	Adequate Precision		45.390	PRESS	223.60	Adequate Precision		67.538

*SS* sum square, *d.f.* degree of freedom, *MS* mean square, *S.D.* standard deviation, *C.V.* coefficient of variation, R^2^ coefficient of determination

To evaluate the performance of optimal levels of the five medium components on RP yields, immobilized cultures (spores and mycelia) were cultivated separately under the optimized levels. In the case of immobilized spores, the predicted RP yield at the optimum levels was 65.651 CV mL^−1^ while the actual experimental yield was 63.119 CV mL^−1^. In the case of immobilized mycelia, the predicted RP yield at the optimum levels was 86.140 CV mL^−1^ while the actual experimental yield was 82.895 CV mL^−1^. The use of RSM led to an intensification of the RP yield of immobilized spores and mycelia by approximately 6 times that of the unoptimized cultures. The results of the experiments from the two model validations were coincident with the estimated values, confirming the high accuracy of both the immobilized spores and mycelia models. In the literature, the optimal sources of nitrogen, carbon, and micronutrients for pigment production by different *Monascus* species were strain-dependent [22, 44–46]. Carbon and nitrogen play a crucial role in cellular metabolism and influence growth and pigment production [47]. In agreement with our results, several reports concluded that glucose is the best substrate for maximum pigment production by different *Monascus* species [7]. Moreover, pigments from cultures of several *Monascus* fungi originate from medium-chain fatty acids. The fatty acid metabolic pathway synthesizes such chain and binds to the chromophore structure through a transesterification reaction which results in the formation of orange pigment. The RP is produced by the reaction of the orange pigment with compounds that contain NH_3_ and NH_2_, such as MSG [48, 49]. In the literature, several *Monascus* strains were reported with diverse productivities. Maximum RP production (22.25 UA_500_) was achieved using MSG [50]. The maximum RP yield (20.44 U abs_500 nm_/mg dfb) was achieved after 12 days of incubation [51]. Moreover, the average RP production recorded a 0.072 AU_510_ h^−1^ in glucose media [52]. The specific productivity of RP of 32.5 OD_500_ g DCW^−1^ h^−1^ was achieved under the optimum culture conditions of batch fermentation [21]. The maximal value of 108.02 ODU/ml was recorded from *M. purpureus* ATCC1643630 cultures [26].

### Cell recycle batch fermentation of RP

Here, we describe the availability of repeated utilization of the immobilized spores and mycelia of *M. ruber* SRZ112—*m22* in a semi-continuous mode of RP production for up to eight successive cycles. Two forms of immobilization were tested viz., spores immobilized cultures and mycelium immobilized cultures. Cultures were grown in 50 mL modified Lin’s medium (pH 6.0) inoculated with 50 calcium alginate beads and incubated at 120 rpm and 25ºC for 10 days. As shown in Table 6, RP yields obtained in all eight cycles were 241.04 CV mL^−1^ (immobilized spores) and 309.17 CV mL^−1^ (immobilized mycelia). For both cultures, the highest RP yields (67.86 ± 1.04 CV mL^−1^ and 89.01 ± 4.82 CV mL^−1^, respectively) were achieved in the second cycle of fermentation. After this, a gradual decrease in RP yield was observed reaching the lowest concentration at the eighth cycle of fermentation. The significant decrease in recorded RP yields from the third cycle to the eighth cycle is apparently because of the aging of the immobilized cells. After the third cycle of fermentation, the ability to metabolize substrates by the immobilized cells was decreased. Furthermore, the cell decay was likely due to aging. In accordance with our results, the alginate-immobilized mycelium of *M. purpureus* C322 was used in an extended repeated batch process (nine batches, 55 days) [35]. The authors further explained that during the first two cycles pigment production was very high then, it began to decrease till the seventh cycle. In contrast, the maximum concentration of several products was achieved in the first cycle including cyclosporin A [53], mycophenolic acid [9], and paclitaxel [12, 20]. Our data showed that the three first cycles are best for the highest productivity by both cultures. Accordingly, we suggest conducting the semi-continuous mode using mycelium-immobilized cultures till the fourth cycle.

**Table 6:** Dry biomass (g L^−1^) RP production (CV mL^−1^ culture filtrate) by immobilized spores and mycelia of *M. ruber* SRZ112–*m22* in calcium alginate beads grown for six different fermentation cycles

Number of fermentation cycles	Immobilized spores		Immobilized mycelia	
	Dry biomass (g L^−1^)	RP yield (CV mL^−1^)	Dry biomass (g L^−1^)	RP yield (CV mL^−1^)
1	–	65.01 ± 2.28^a^	–	85.74 ± 3.56^a^
2	–	67.86 ± 1.04^a^	–	89.01 ± 4.82^a^
3	–	66.73 ± 2.87^a^	–	78.77 ± 2.99^b^
4	–	30.05 ± 1.25^b^	–	47.55 ± 5.41^c^
5	–	6.65 ± 3.25^c^	–	4.76 ± 2.54^d^
6	–	3.89 ± 2.52^c^	–	2.79 ± 3.82^d^
7	–	0.76 ± 0.95^d^	–	0.55 ± 1.01^e^
8	14.76 ± 0.98	0.09 ± 0.001^e^	16.01 ± 0.57	0.003 ± 0.002^f^
**Total**	**14.76**	**241.04**	**16.01**	**309.17**

Cultures were grown in 50 mL modified Lin’s medium (pH 6.0) inoculated with 50 calcium alginate beads and incubated at 120 rpm and 25ºC for 10 days. Calculated mean is for triplicate measurements from two independent experiments. The means with different superscripts in the same column are considered statistically different (LSD test, *P* ≤ 0.05)

## Conclusions

A stable mutant strain with improved RP productivity was developed using gamma irradiation. This mutant was then employed in the immobilization technique using various entrapment carriers. Then, the optimal medium for maximum RP production by immobilized cultures of this mutant was developed using RSM. Eight different fermentation media were tested and the maximum RP yield (10.51 ± 1.01 CV mL^−1^ for immobilized spores and 12.54 ± 1.08 CV mL^−1^ for immobilized mycelia) was achieved using modified Lin’s medium. Following RSM, maximum RP yields (65.651 CV mL^−1^ for spores and 86.140 for mycelia CV mL^−1^) were attained when the concentration of glucose, glucose, MSG, KH_2_PO_4_, MgSO_4_⋅7H_2_O, FeSO_4_⋅7H_2_O were 23.54, 28.77, 1.69, 1.047, and 0.545 g L^−1^, respectively. Finally, these immobilized cultures were successfully utilized for RP production using a cell recycles batch fermentation dramatically intensifying the highest RP yield and recording 309.17 CV mL^−1^, a significant increase compared to the free cultures. Accordingly, the presented research greatly recommends the developed mutant *M. ruber* SRZ112—*m22* as a promising biofactory of natural pigments.

## Data Availability

All data generated or analyzed during this study are included in this published article.

## Data Availability

No datasets were generated or analysed during the current study.
